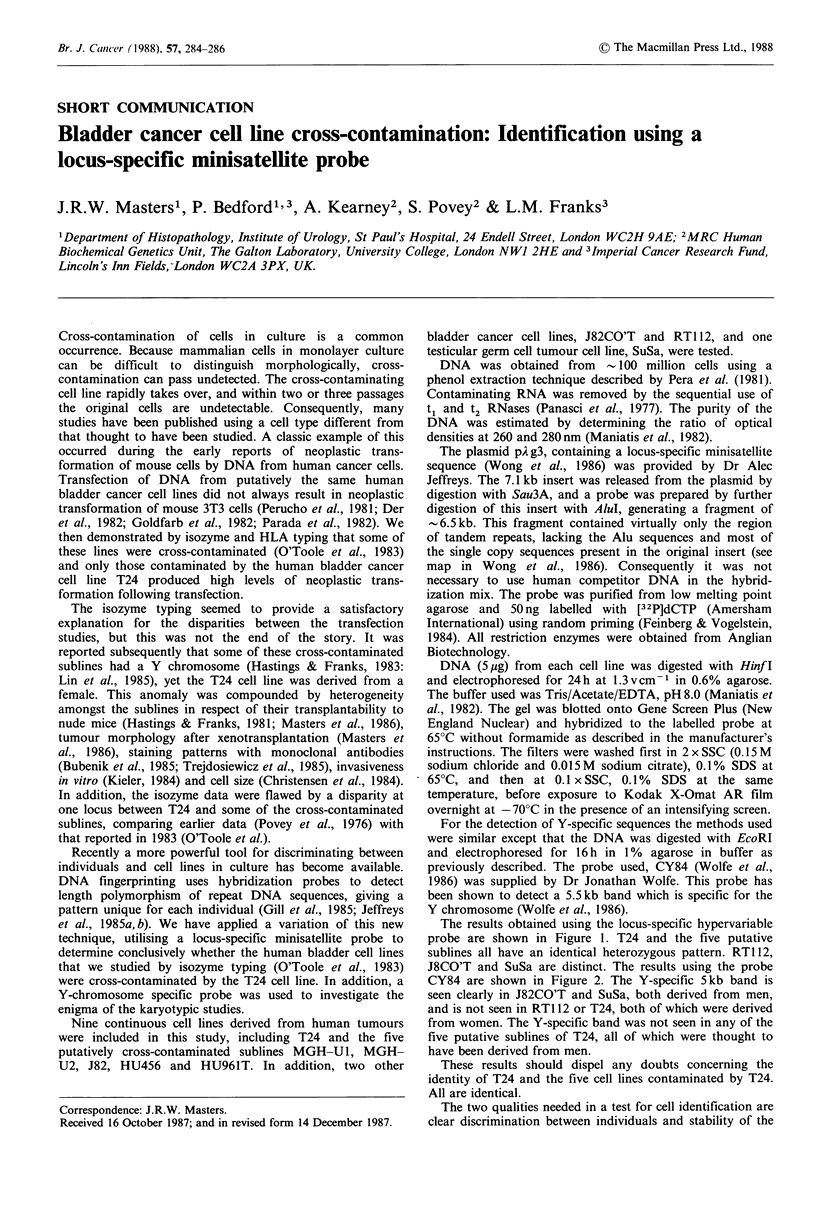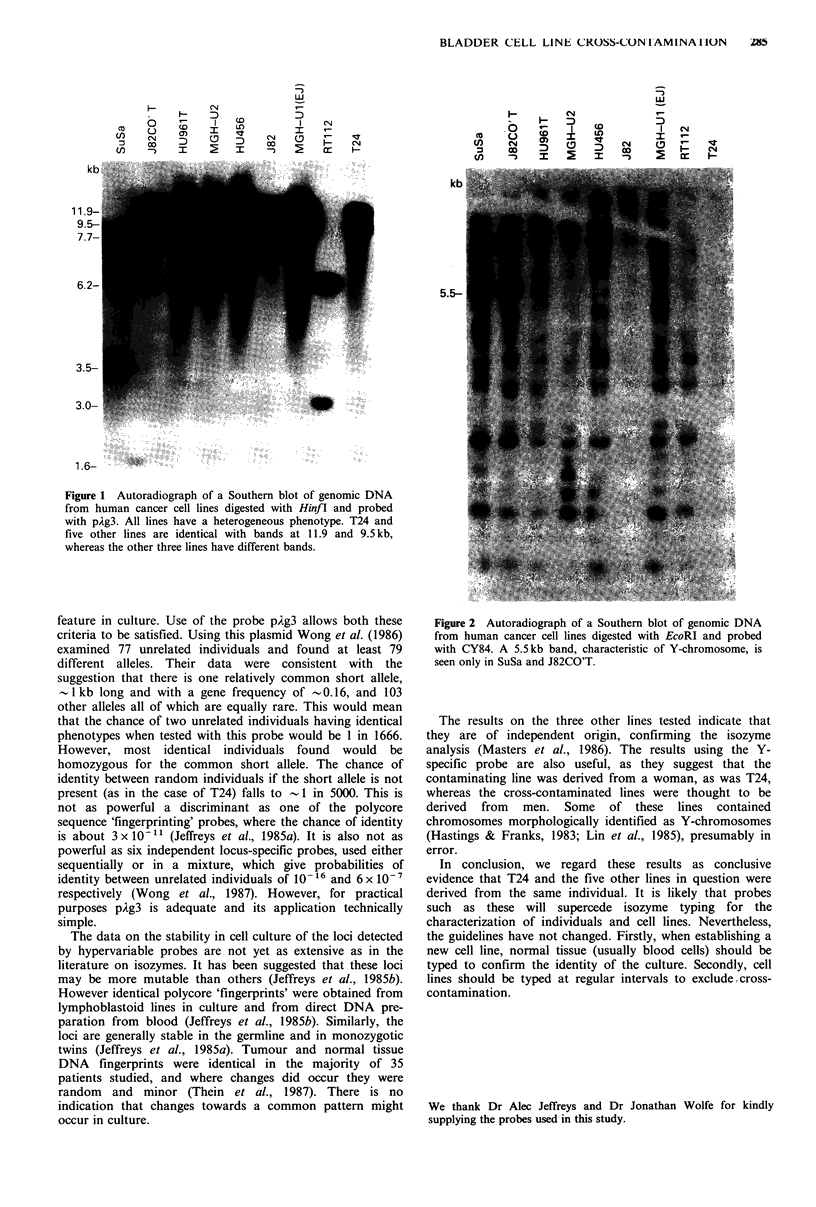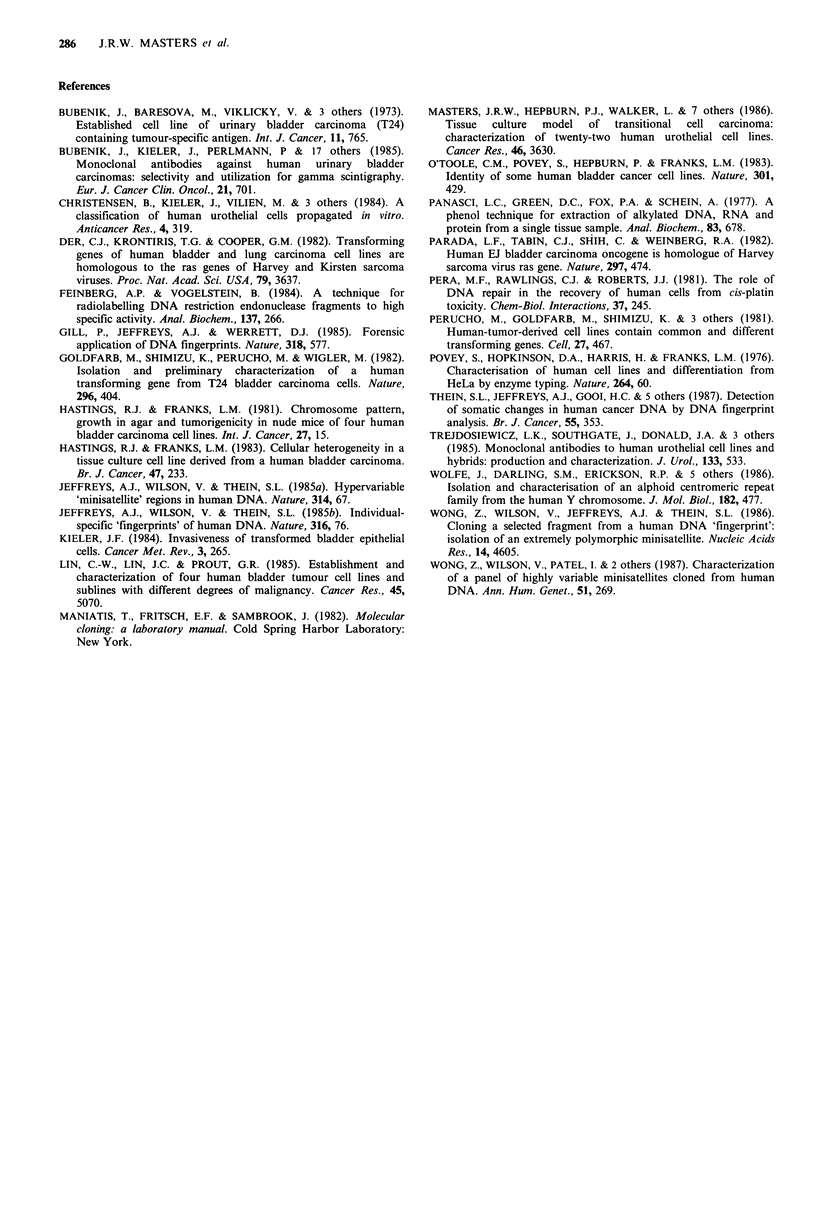# Bladder cancer cell line cross-contamination: identification using a locus-specific minisatellite probe.

**DOI:** 10.1038/bjc.1988.61

**Published:** 1988-03

**Authors:** J. R. Masters, P. Bedford, A. Kearney, S. Povey, L. M. Franks

**Affiliations:** Department of Histopathology, St Paul's Hospital, London, UK.

## Abstract

**Images:**


Br. J. Cancer (1988), 57, 284–286 © The Macmillan Press Ltd., 1988

SHORT COMMUNICATION

### Bladder cancer cell line cross-contamination: Identification using a locus-specific minisatellite probe

J.R.W. Masters[1], P. Bedford[1,3], A. Kearney[2], S. Povey[2] & L.M. Franks[3]

[1]*Department of Histopathology, Institute of Urology, St Paul's Hospital, 24 Endell Street, London WC2H 9AE;* [2]*MRC Human Biochemical Genetics Unit, The Galton Laboratory, University College, London NW1 2HE and* [3]*Imperial Cancer Research Fund, Lincoln's Inn Fields, London WC2A 3PX, UK.*

Cross-contamination of cells in culture is a common occurrence. Because mammalian cells in monolayer culture can be difficult to distinguish morphologically, cross-contamination can pass undetected. The cross-contaminating cell line rapidly takes over, and within two or three passages the original cells are undetectable. Consequently, many studies have been published using a cell type different from that thought to have been studied. A classic example of this occurred during the early reports of neoplastic transformation of mouse cells by DNA from human cancer cells. Transfection of DNA from putatively the same human bladder cancer cell lines did not always result in neoplastic transformation of mouse 3T3 cells (Perucho *et al.*, 1981; Der *et al.*, 1982; Goldfarb *et al.*, 1982; Parada *et al.*, 1982). We then demonstrated by isozyme and HLA typing that some of these lines were cross-contaminated (O'Toole *et al.*, 1983) and only those contaminated by the human bladder cancer cell line T24 produced high levels of neoplastic transformation following transfection.

The isozyme typing seemed to provide a satisfactory explanation for the disparities between the transfection studies, but this was not the end of the story. It was reported subsequently that some of these cross-contaminated sublines had a Y chromosome (Hastings & Franks, 1983: Lin *et al.*, 1985), yet the T24 cell line was derived from a female. This anomaly was compounded by heterogeneity amongst the sublines in respect of their transplantability to nude mice (Hastings & Franks, 1981; Masters *et al.*, 1986), tumour morphology after xenotransplantation (Masters *et al.*, 1986), staining patterns with monoclonal antibodies (Bubenik *et al.*, 1985; Trejdosiewicz *et al.*, 1985), invasiveness *in vitro* (Kieler, 1984) and cell size (Christensen *et al.*, 1984). In addition, the isozyme data were flawed by a disparity at one locus between T24 and some of the cross-contaminated sublines, comparing earlier data (Povey *et al.*, 1976) with that reported in 1983 (O'Toole *et al.*).

Recently a more powerful tool for discriminating between individuals and cell lines in culture has become available. DNA fingerprinting uses hybridization probes to detect length polymorphism of repeat DNA sequences, giving a pattern unique for each individual (Gill *et al.*, 1985; Jeffreys *et al.*, 1985*a,b*). We have applied a variation of this new technique, utilising a locus-specific minisatellite probe to determine conclusively whether the human bladder cell lines that we studied by isozyme typing (O'Toole *et al.*, 1983) were cross-contaminated by the T24 cell line. In addition, a Y-chromosome specific probe was used to investigate the enigma of the karyotypic studies.

Nine continuous cell lines derived from human tumours were included in this study, including T24 and the five putatively cross-contaminated sublines MGH-U1, MGH-U2, J82, HU456 and HU961T. In addition, two other bladder cancer cell lines, J82CO'T and RT112, and one testicular germ cell tumour cell line, SuSa, were tested.

DNA was obtained from ~100 million cells using a phenol extraction technique described by Pera *et al.* (1981). Contaminating RNA was removed by the sequential use of $t_1$ and $t_2$ RNases (Panasci *et al.*, 1977). The purity of the DNA was estimated by determining the ratio of optical densities at 260 and 280 nm (Maniatis *et al.*, 1982).

The plasmid pλg3, containing a locus-specific minisatellite sequence (Wong *et al.*, 1986) was provided by Dr Alec Jeffreys. The 7.1 kb insert was released from the plasmid by digestion with *Sau*3A, and a probe was prepared by further digestion of this insert with *Alu*I, generating a fragment of ~6.5 kb. This fragment contained virtually only the region of tandem repeats, lacking the Alu sequences and most of the single copy sequences present in the original insert (see map in Wong *et al.*, 1986). Consequently it was not necessary to use human competitor DNA in the hybridization mix. The probe was purified from low melting point agarose and 50 ng labelled with [$^{32}$P]dCTP (Amersham International) using random priming (Feinberg & Vogelstein, 1984). All restriction enzymes were obtained from Anglian Biotechnology.

DNA (5 μg) from each cell line was digested with *Hinf*I and electrophoresed for 24 h at 1.3 v cm$^{-1}$ in 0.6% agarose. The buffer used was Tris/Acetate/EDTA, pH 8.0 (Maniatis *et al.*, 1982). The gel was blotted onto Gene Screen Plus (New England Nuclear) and hybridized to the labelled probe at 65°C without formamide as described in the manufacturer's instructions. The filters were washed first in 2 × SSC (0.15 M sodium chloride and 0.015 M sodium citrate), 0.1% SDS at 65°C, and then at 0.1 × SSC, 0.1% SDS at the same temperature, before exposure to Kodak X-Omat AR film overnight at −70°C in the presence of an intensifying screen.

For the detection of Y-specific sequences the methods used were similar except that the DNA was digested with *Eco*RI and electrophoresed for 16 h in 1% agarose in buffer as previously described. The probe used, CY84 (Wolfe *et al.*, 1986) was supplied by Dr Jonathan Wolfe. This probe has been shown to detect a 5.5 kb band which is specific for the Y chromosome (Wolfe *et al.*, 1986).

The results obtained using the locus-specific hypervariable probe are shown in Figure 1. T24 and the five putative sublines all have an identical heterozygous pattern. RT112, J8CO'T and SuSa are distinct. The results using the probe CY84 are shown in Figure 2. The Y-specific 5 kb band is seen clearly in J82CO'T and SuSa, both derived from men, and is not seen in RT112 or T24, both of which were derived from women. The Y-specific band was not seen in any of the five putative sublines of T24, all of which were thought to have been derived from men.

These results should dispel any doubts concerning the identity of T24 and the five cell lines contaminated by T24. All are identical.

The two qualities needed in a test for cell identification are clear discrimination between individuals and stability of the

Correspondence: J.R.W. Masters.
Received 16 October 1987; and in revised form 14 December 1987.

**Figure 1** Autoradiograph of a Southern blot of genomic DNA from human cancer cell lines digested with *Hinf*I and probed with p$\lambda$g3. All lines have a heterogeneous phenotype. T24 and five other lines are identical with bands at 11.9 and 9.5 kb, whereas the other three lines have different bands.

**Figure 2** Autoradiograph of a Southern blot of genomic DNA from human cancer cell lines digested with *Eco*RI and probed with CY84. A 5.5 kb band, characteristic of Y-chromosome, is seen only in SuSa and J82CO'T.

feature in culture. Use of the probe p$\lambda$g3 allows both these criteria to be satisfied. Using this plasmid Wong *et al.* (1986) examined 77 unrelated individuals and found at least 79 different alleles. Their data were consistent with the suggestion that there is one relatively common short allele, ~1 kb long and with a gene frequency of ~0.16, and 103 other alleles all of which are equally rare. This would mean that the chance of two unrelated individuals having identical phenotypes when tested with this probe would be 1 in 1666. However, most identical individuals found would be homozygous for the common short allele. The chance of identity between random individuals if the short allele is not present (as in the case of T24) falls to ~1 in 5000. This is not as powerful a discriminant as one of the polycore sequence 'fingerprinting' probes, where the chance of identity is about $3 \times 10^{-11}$ (Jeffreys *et al.*, 1985*a*). It is also not as powerful as six independent locus-specific probes, used either sequentially or in a mixture, which give probabilities of identity between unrelated individuals of $10^{-16}$ and $6 \times 10^{-7}$ respectively (Wong *et al.*, 1987). However, for practical purposes p$\lambda$g3 is adequate and its application technically simple.

The data on the stability in cell culture of the loci detected by hypervariable probes are not yet as extensive as in the literature on isozymes. It has been suggested that these loci may be more mutable than others (Jeffreys *et al.*, 1985*b*). However identical polycore 'fingerprints' were obtained from lymphoblastoid lines in culture and from direct DNA preparation from blood (Jeffreys *et al.*, 1985*b*). Similarly, the loci are generally stable in the germline and in monozygotic twins (Jeffreys *et al.*, 1985*a*). Tumour and normal tissue DNA fingerprints were identical in the majority of 35 patients studied, and where changes did occur they were random and minor (Thein *et al.*, 1987). There is no indication that changes towards a common pattern might occur in culture.

The results on the three other lines tested indicate that they are of independent origin, confirming the isozyme analysis (Masters *et al.*, 1986). The results using the Y-specific probe are also useful, as they suggest that the contaminating line was derived from a woman, as was T24, whereas the cross-contaminated lines were thought to be derived from men. Some of these lines contained chromosomes morphologically identified as Y-chromosomes (Hastings & Franks, 1983; Lin *et al.*, 1985), presumably in error.

In conclusion, we regard these results as conclusive evidence that T24 and the five other lines in question were derived from the same individual. It is likely that probes such as these will supercede isozyme typing for the characterization of individuals and cell lines. Nevertheless, the guidelines have not changed. Firstly, when establishing a new cell line, normal tissue (usually blood cells) should be typed to confirm the identity of the culture. Secondly, cell lines should be typed at regular intervals to exclude cross-contamination.

We thank Dr Alec Jeffreys and Dr Jonathan Wolfe for kindly supplying the probes used in this study.

### References

BUBENIK, J., BARESOVA, M., VIKLICKY, V. & 3 others (1973). Established cell line of urinary bladder carcinoma (T24) containing tumour-specific antigen. *Int. J. Cancer*, **11**, 765.

BUBENIK, J., KIELER, J., PERLMANN, P & 17 others (1985). Monoclonal antibodies against human urinary bladder carcinomas: selectivity and utilization for gamma scintigraphy. *Eur. J. Cancer Clin. Oncol.*, **21**, 701.

CHRISTENSEN, B., KIELER, J., VILIEN, M. & 3 others (1984). A classification of human urothelial cells propagated *in vitro*. *Anticancer Res.*, **4**, 319.

DER, C.J., KRONTIRIS, T.G. & COOPER, G.M. (1982). Transforming genes of human bladder and lung carcinoma cell lines are homologous to the ras genes of Harvey and Kirsten sarcoma viruses. *Proc. Nat. Acad. Sci. USA*, **79**, 3637.

FEINBERG, A.P. & VOGELSTEIN, B. (1984). A technique for radiolabelling DNA restriction endonuclease fragments to high specific activity. *Anal. Biochem.*, **137**, 266.

GILL, P., JEFFREYS, A.J. & WERRETT, D.J. (1985). Forensic application of DNA fingerprints. *Nature*, **318**, 577.

GOLDFARB, M., SHIMIZU, K., PERUCHO, M. & WIGLER, M. (1982). Isolation and preliminary characterization of a human transforming gene from T24 bladder carcinoma cells. *Nature*, **296**, 404.

HASTINGS, R.J. & FRANKS, L.M. (1981). Chromosome pattern, growth in agar and tumorigenicity in nude mice of four human bladder carcinoma cell lines. *Int. J. Cancer*, **27**, 15.

HASTINGS, R.J. & FRANKS, L.M. (1983). Cellular heterogeneity in a tissue culture cell line derived from a human bladder carcinoma. *Br. J. Cancer*, **47**, 233.

JEFFREYS, A.J., WILSON, V. & THEIN, S.L. (1985*a*). Hypervariable 'minisatellite' regions in human DNA. *Nature*, **314**, 67.

JEFFREYS, A.J., WILSON, V. & THEIN, S.L. (1985*b*). Individual-specific 'fingerprints' of human DNA. *Nature*, **316**, 76.

KIELER, J.F. (1984). Invasiveness of transformed bladder epithelial cells. *Cancer Met. Rev.*, **3**, 265.

LIN, C.-W., LIN, J.C. & PROUT, G.R. (1985). Establishment and characterization of four human bladder tumour cell lines and sublines with different degrees of malignancy. *Cancer Res.*, **45**, 5070.

MANIATIS, T., FRITSCH, E.F. & SAMBROOK, J. (1982). *Molecular cloning: a laboratory manual*. Cold Spring Harbor Laboratory: New York.

MASTERS, J.R.W., HEPBURN, P.J., WALKER, L. & 7 others (1986). Tissue culture model of transitional cell carcinoma: characterization of twenty-two human urothelial cell lines. *Cancer Res.*, **46**, 3630.

O'TOOLE, C.M., POVEY, S., HEPBURN, P. & FRANKS, L.M. (1983). Identity of some human bladder cancer cell lines. *Nature*, **301**, 429.

PANASCI, L.C., GREEN, D.C., FOX, P.A. & SCHEIN, A. (1977). A phenol technique for extraction of alkylated DNA, RNA and protein from a single tissue sample. *Anal. Biochem.*, **83**, 678.

PARADA, L.F., TABIN, C.J., SHIH, C. & WEINBERG, R.A. (1982). Human EJ bladder carcinoma oncogene is homologue of Harvey sarcoma virus ras gene. *Nature*, **297**, 474.

PERA, M.F., RAWLINGS, C.J. & ROBERTS, J.J. (1981). The role of DNA repair in the recovery of human cells from *cis*-platin toxicity. *Chem-Biol. Interactions*, **37**, 245.

PERUCHO, M., GOLDFARB, M., SHIMIZU, K. & 3 others (1981). Human-tumor-derived cell lines contain common and different transforming genes. *Cell*, **27**, 467.

POVEY, S., HOPKINSON, D.A., HARRIS, H. & FRANKS, L.M. (1976). Characterisation of human cell lines and differentiation from HeLa by enzyme typing. *Nature*, **264**, 60.

THEIN, S.L., JEFFREYS, A.J., GOOI, H.C. & 5 others (1987). Detection of somatic changes in human cancer DNA by DNA fingerprint analysis. *Br. J. Cancer*, **55**, 353.

TREJDOSIEWICZ, L.K., SOUTHGATE, J., DONALD, J.A. & 3 others (1985). Monoclonal antibodies to human urothelial cell lines and hybrids: production and characterization. *J. Urol.*, **133**, 533.

WOLFE, J., DARLING, S.M., ERICKSON, R.P. & 5 others (1986). Isolation and characterisation of an alphoid centromeric repeat family from the human Y chromosome. *J. Mol. Biol.*, **182**, 477.

WONG, Z., WILSON, V., JEFFREYS, A.J. & THEIN, S.L. (1986). Cloning a selected fragment from a human DNA 'fingerprint': isolation of an extremely polymorphic minisatellite. *Nucleic Acids Res.*, **14**, 4605.

WONG, Z., WILSON, V., PATEL, I. & 2 others (1987). Characterization of a panel of highly variable minisatellites cloned from human DNA. *Ann. Hum. Genet.*, **51**, 269.